# Overexpression of the transcription factor RAP2.6 leads to enhanced callose deposition in syncytia and enhanced resistance against the beet cyst nematode *Heterodera schachtii* in Arabidopsis roots

**DOI:** 10.1186/1471-2229-13-47

**Published:** 2013-03-19

**Authors:** Muhammad Amjad Ali, Amjad Abbas, David P Kreil, Holger Bohlmann

**Affiliations:** 1 Division of Plant Protection, Department of Crop Sciences, University of Natural Resources and Life Sciences Vienna, UFT Tulln, Konrad Lorenz Str. 24, 3430, Tulln, Austria; 2 Current address: Department of Bioinformatics and Biotechnology, GC University, Faisalabad, Pakistan; 3 Chair of Bioinformatics, Department of Biotechnology, University of Natural Resources and Life Sciences Vienna, Vienna, Austria; 4 School of Life Sciences, University of Warwick, Warwick, UK

## Abstract

**Background:**

Cyst nematodes invade the roots of their host plants as second stage juveniles and induce a syncytium which is their source of nutrients throughout their life. A transcriptome analysis of syncytia induced by the beet cyst nematode *Heterodera schachtii* in Arabidopsis roots has shown that gene expression in the syncytium is different from that of the root with thousands of genes upregulated or downregulated. Among the downregulated genes are many which code for defense-related proteins. One gene which is strongly downregulated codes for the ethylene response transcription factor RAP2.6. The genome of Arabidopsis contains 122 ERF transcription factor genes which are involved in a variety of developmental and stress responses.

**Results:**

Expression of *RAP2.6* was studied with RT-PCR and a promoter::GUS line. During normal growth conditions the gene was expressed especially in roots and stems. It was inducible by *Pseudomonas syringae* but downregulated in syncytia from a very early time point on. Overexpression of the gene enhanced the resistance against *H. schachtii* which was seen by a lower number of nematodes developing on these plants as well as smaller syncytia and smaller female nematodes. A T-DNA mutant had a reduced *RAP2.6* transcript level but this did not further increase the susceptibility against *H. schachtii*. Neither overexpression lines nor mutants had an effect on *P. syringae*. Overexpression of RAP2.6 led to an elevated expression of JA-responsive genes during early time points after infection by *H. schachtii*. Syncytia developing on overexpression lines showed enhanced deposition of callose.

**Conclusions:**

Our results showed that *H. schachtii* infection is accompanied by a downregulation of *RAP2.6*. It seems likely that the nematodes use effectors to actively downregulate the expression of this and other defense-related genes to avoid resistance responses of the host plant. Enhanced resistance of RAP2.6 overexpression lines seemed to be due to enhanced callose deposition at syncytia which might interfere with nutrient import into syncytia.

## Background

Nematodes are multicellular unsegmented soft-bodied worms and belong to the phylum Nematoda. They are ubiquitous in nature and can be found everywhere ranging from the sediments of the oceans to high mountains and in a variety of climates [1]. Plant parasitic nematodes are obligate biotrophic parasites generally attacking the roots of many plant species. They have a wide host range and can have adverse effects on the yield of crop plants by damaging the crops either directly or as virus vectors. The worldwide annual crop losses caused by plant parasitic nematodes have been estimated at 157 billion dollars [2].

Several economically important species are pathogens of different crop plants and the cyst and root-knot nematodes within the family *Heteroderidae* are among the most important. They are obligate endoparasites of plant roots which they enter as second stage juveniles (J2 larvae) and establish specialized feeding structures [3,4]. Root-knot nematodes belonging to the genus *Meloidogyne* induce a feeding structure comprised of several giant cells [5]. Cyst nematodes of the genera *Heterodera* and *Globodera* hatch from eggs as J2 larvae and pierce the host plant roots by continuously striking their stylet just around the root elongation zone. After entering the root, they migrate intracellularly through the root cortex to find the vascular cylinder. When the nematodes reach the vascular bundle, they initiate a specialized feeding site called a syncytium [6]. The syncytium originates from a single root cell (ISC, initial syncytial cell) which expands by incorporating up to several hundred adjacent cells by local cell wall dissolution. It has been shown that plant encoded cell wall modifying and degrading enzymes such as expansins, pectinases, and cellulases are involved in this process [7-11]. The syncytium becomes the only food source for the nematodes as they develop through subsequent sedentary life stages [12,13]. Adult male cyst nematodes become mobile again and leave their feeding site to mate with females while females remain attached with their syncytium. After mating, the female cyst nematode carries on feeding but dies after the completion of egg development, leaving several hundred eggs contained within its enlarged body. The outer layer of the female subsequently hardens to form a cyst, which protects the eggs until infective J2 hatch under favorable conditions [3].

The establishment of the syncytium from the ISC inside the vascular cylinder is most likely commenced through secretions of the nematode and a coordinated expression of plant genes [9-11,14,15]. Recently, we have performed a transcriptome analysis of 5 and 15 dpi (days post infection) syncytia induced by *H. schachtii* in Arabidopsis roots which revealed that 34.2% out of a total of 21,138 Arabidopsis genes were differentially expressed as compared to uninfected control root sections [16]. Of these differentially expressed genes, 18.4% (3893) were upregulated while 15.8% (3338) were downregulated. Upregulated genes included for instance those coding for expansins, cellulases, and pectate lyases [9-11] which are involved in cell wall degradation and genes coding for myo-inositol oxygenases [17]. On the other hand, genes which were strongly repressed after nematode infection were related to defense responses of the plant [16]. One strongly downregulated group comprised for instance genes coding for peroxidases and out of 100 differentially expressed genes with the strongest decrease in expression, 14 were peroxidases [16].

Another gene which was significantly downregulated in syncytia as compared to control root sections was the *RAP2.6* gene [16]. Members of this family of proteins contain the APETALA2 (AP2) domain and were first defined as a family encoded by 12 genes in Arabidopsis. APETALA2 was found to be involved in the control of Arabidopsis flower and seed development and encodes a putative transcription factor that is distinguished by a novel DNA binding motif referred to as the AP2 domain [18]. Related proteins were originally identified as transcriptional regulators that function downstream of ethylene signaling [19]. All these and other proteins are now included in the AP2/ERF superfamily which has 147 members in Arabidopsis [20]. The largest group of these includes the ethylene response factors (ERFs) with 122 members. This group contains the originally described RAP2 proteins in different subgroups.

The *RAP2.6* gene has been reported to respond to various biotic and abiotic stresses indicating its role in the regulation of these stresses. *RAP2.6* was found to be involved in the *Arabidopsis* response to abscisic acid (ABA), wounding, jasmonic acid (JA), salt, cold, and osmotic stresses [21-24]. The activation of *RAP2.6* in response to type III secretions of virulent and avirulent strains of *Pseudomonas syringae* was reported to be dependent on *Coi1* (coronatine-insenstive 1) by [25]. Similarly, RAP2.6 was identified as a *Coi1*- dependent JA-inducible transcription factor [26]. Using a *RAP2.6::LUC* reporter gene, it was found that *RAP2.6* was induced by the virulent bacterium *P. syringae* pv *tomato* but not by the non-adapted bacterium *P. syringae* pv *phaseolicola*[27]. It is well known that *P. syringae* uses coronatin to induce the JA pathway in the host plant to suppress salicylic acid (SA) dependent resistance [28]. *RAP2.6* was also highly upreguled after 24 h in response to the diamond black moth [29]. All these reports indicate that the *RAP2.6* gene is involved in the JA response. Indeed, this gene was among 14 AP2/ERF genes that were found in a screening for JA-inducible ERF transcription factors [30] which also included ORA59 [31]. Since *RAP2.6* was one of the most strongly downregulated genes in syncytia [16] we have studied this gene in more detail. We reasoned that this gene might be downregulated by *H. schachtii* to avoid a plant resistance response. We have therefore tested if overexpression of the *RAP2.6* gene might lead to higher resistance against *H. schachtii*.

## Results

### Expression of the ERF gene family in syncytia

We recently performed a transcriptome analysis of syncytia induced by *H. schachtii* in Arabidopsis roots [16]. The data from this analysis were used to specifically analyse the expression of the ERF genes in syncytia. The ERF family contains 122 members of which 105 are included on the Arabidopsis GeneChip. Our analysis (Table 1 and Additional file 1) indicated that only 7 of these genes showed a significant upregulation in syncytia as compared to control root sections while 32 showed a significant downregulation (false discovery rate < 5%). Comparing 15 dpi syncytia with 5 dpi syncytia showed that 7 genes were significantly higher expressed in 15 dpi syncytia compared to 5 dpi syncytia (Additional file 2). The genes that showed the strongest downregulation in syncytia were *At5g25810* (*TNY*), *At1g78080* (*RAP2.4*), *At2g20880*, *At3g50260* (*CEJ1* or *DEAR1*[32]) and *At1g43160* (*RAP2.6*), which was the most suppressed gene in this family. Because several reports found that it is involved in plant resistance and the GeneChip data showed the strongest downregulation for *RAP2.6* (expression level 0.91 in syncytia and 9.97 in control root sections), we studied the expression of this gene in detail by using GUS analysis and qRT-PCR in syncytia.

**Table 1:** **Expression of *ERF *genes in syncytia and control root segments according to GeneChip data**

**ID**	**Gene**	**Syn**	**Root**	**Syn vs root**	**q**
***At1g43160***	***RAP2.6***	**0.91**	**9.97**	**−9.06**	**9.8E-8**
***At3g50260***	***CEJ1/ DEAR1***	**3.78**	**9.89**	**−6.11**	**7.1E-5**
***At2g20880***	***AtERF53***	**2.13**	**7.73**	**−5.61**	**9.8E-8**
***At1g78080***	***WIND1/RAP2.4***	**3.35**	**8.43**	**−5.08**	**1.0E-5**
***At5g25810***	***TINY***	**2.08**	**6.33**	**−4.24**	**8.2E-7**
***At5g05410***	***DREB2A***	**5.67**	**9.12**	**−3.45**	**0.10%**
***At1g22190***	***WIND2***	**4.31**	**7.54**	**−3.23**	**7.3E-5**
***At1g53910***	***RAP2.12***	**4.33**	**6.50**	**−2.17**	**3.0%**
***At5g44210***	***AtERF9***	**3.20**	**5.34**	**−2.14**	**0.44%**
***At1g06160***	***ORA59***	**3.25**	**5.37**	**−2.12**	**0.04%**
***At4g17490***	***AtERF6***	**4.12**	**6.21**	**−2.08**	**1.7%**
***At4g39780***		**3.71**	**5.68**	**−1.97**	**0.07%**
***At5g19790***	***RAP2.11***	**3.59**	**5.55**	**−1.96**	**0.01%**
***At2g23340***		**9.41**	**11.26**	**−1.85**	**2.8%**
***At5g61590***		**5.81**	**7.63**	**−1.82**	**1.7%**
***At5g61600***		**5.67**	**7.41**	**−1.74**	**1.5%**
***At5g47230***	***AtERF5***	**3.66**	**5.16**	**−1.50**	**0.53%**
***At3g20310***	***AtERF7***	**5.64**	**7.05**	**−1.41**	**2.9%**
***At1g64380***		**2.25**	**3.58**	**−1.34**	**1.4%**
***At4g36900***	***RAP2.10***	**6.29**	**7.41**	**−1.12**	**3.0%**
*At5g67190*		4.82	5.93	−1.11	41.7%
***At2g44940***		**4.61**	**5.70**	**−1.09**	**0.61%**
***At4g16750***		**2.80**	**3.83**	**−1.03**	**0.04%**
*At1g72360*		8.47	9.50	−1.02	14.2%
***At5g07580***		**5.65**	**6.63**	**−0.98**	**0.10%**
***At4g17500***	***AtERF1***	**5.75**	**6.73**	**−0.98**	**0.89%**
*At3g14230*	*RAP2.2*	4.88	5.76	−0.88	23.4%
***At2g25820***		**2.70**	**3.54**	**−0.84**	**2.7%**
***At4g25480***		**3.04**	**3.87**	**−0.83**	**2.3%**
***At3g61630***		**2.18**	**2.95**	**−0.77**	**0.61%**
***At5g18560***		**3.10**	**3.86**	**−0.76**	**0.81%**
*At1g22985*		6.17	6.91	−0.74	9.6%
*At5g18450*		4.03	4.76	−0.73	7.7%
***At3g15210***	***AtERF4/RAP2.5***	**5.14**	**5.81**	**−0.67**	**2.3%**
***At5g51990***	***CBF4/DREB1D***	**3.20**	**3.74**	**−0.55**	**2.9%**
*At1g53170*	*AtERF8*	4.33	4.75	−0.42	13.9%
*At5g67000*		4.04	4.45	−0.41	44.2%
***At5g13910***	***LEP***	**2.96**	**3.35**	**−0.39**	**4.1%**
***At1g46768***	***RAP2.1***	**3.70**	**4.08**	**−0.38**	**2.7%**
*At2g46310*		3.41	3.74	−0.34	13.5%
*At2g40220*	*ABI4*	3.27	3.58	−0.31	7.7%
*At3g23230*	*TDR1*	2.93	3.24	−0.31	30.1%
*At4g25490*	*CBF1/DREB1B*	3.30	3.61	−0.31	8.7%
*At2g31230*	*AtERF15*	3.59	3.87	−0.29	14.6%
*At4g28140*		2.75	3.02	−0.27	16.6%
*At3g60490*		3.71	3.97	−0.25	30.1%
*At4g11140*		2.67	2.91	−0.24	20.2%
*At5g25390*	*SHN3*	3.97	4.20	−0.23	44.2%
*At1g15360*	*WIN1/SHN1*	3.06	3.26	−0.20	30.7%
*At5g65130*	*WIND4*	2.44	2.64	−0.20	14.2%
*At4g18450*		3.34	3.53	−0.19	19.5%
*At5g11590*		2.46	2.65	−0.19	27.2%
*At1g75490*		2.64	2.82	−0.19	38.5%
*At1g44830*		3.14	3.30	−0.16	41.7%
*At1g33760*		2.14	2.29	−0.14	30.7%
*At4g31060*		3.87	3.99	−0.13	80.3%
*At5g67010*		2.62	2.74	−0.12	45.1%
*At1g63030*	*DDF1*	1.97	2.07	−0.10	48.3%
*At1g22810*		2.26	2.35	−0.09	52.8%
*At3g23220*		3.52	3.61	−0.09	52.8%
*At5g43410*		3.93	4.01	−0.08	67.5%
*At1g28160*		2.49	2.55	−0.06	70.3%
*At1g28370*	*AtERF11*	5.00	5.05	−0.05	92.5%
*At2g22200*		2.70	2.75	−0.05	80.3%
*At5g25190*		3.06	3.11	−0.05	80.3%
*At5g07310*		3.11	3.15	−0.04	86.9%
*At1g80580*		3.17	3.21	−0.04	91.2%
*At1g77640*		4.41	4.44	−0.03	91.2%
*At1g12630*		4.85	4.88	−0.03	91.2%
*At3g16280*		3.97	3.99	−0.02	91.2%
*At2g36450*		3.01	3.03	−0.02	91.2%
*At2g35700*		2.78	2.80	−0.02	91.6%
*At1g01250*		3.24	3.24	0.00	99.3%
*At4g25470*	*CBF2/DREB1C*	4.30	4.30	0.01	96.8%
*At1g50640*	*AtERF3*	2.95	2.94	0.01	95.6%
*At3g57600*		2.73	2.70	0.03	91.2%
*At1g12610*		2.58	2.55	0.03	91.2%
*At2g33710*		2.82	2.78	0.04	78.0%
*At3g23240*	*ERF1*	3.49	3.45	0.05	81.8%
*At2g20350*		2.61	2.56	0.05	86.9%
*At1g19210*		3.22	3.14	0.08	73.9%
*At5g53290*		3.45	3.36	0.10	67.4%
*At2g44840*	*AtERF13*	3.75	3.65	0.10	67.0%
*At1g68550*		5.06	4.93	0.13	53.9%
*At1g36060*	*WIND3*	4.21	4.07	0.15	50.5%
*At5g52020*		3.87	3.69	0.18	56.1%
*At1g28360*	*AtERF12*	2.84	2.63	0.21	16.6%
*At4g32800*		3.70	3.48	0.22	14.2%
*At3g11020*	*DREB2B*	4.75	4.51	0.24	25.4%
*At5g47220*	*AtERF2*	4.12	3.87	0.26	18.9%
*At1g25470*		5.06	4.79	0.26	36.0%
*At2g38340*	*DREB2E*	2.86	2.58	0.27	9.6%
*At1g74930*		3.78	3.35	0.43	5.7%
*At4g23750*		3.72	3.28	0.44	14.2%
*At1g71130*		6.75	6.27	0.48	14.2%
*At3g25890*		3.96	3.37	0.59	7.1%
*At5g51190*		5.43	4.82	0.61	11.4%
***At5g61890***		**4.86**	**4.25**	**0.62**	**0.89%**
***At1g71450***		**3.23**	**2.58**	**0.65**	**0.73%**
*At2g47520*		4.36	3.62	0.74	8.3%
***At1g77200***		**3.18**	**2.43**	**0.75**	**0.61%**
***At5g13330***		**6.03**	**5.17**	**0.85**	**2.2%**
***At1g21910***		**4.56**	**3.58**	**0.98**	**1.4%**
***At4g34410***		**4.40**	**2.84**	**1.56**	**0.77%**
***At3g16770***	***AtEBP/RAP2.3***	**11.15**	**9.34**	**1.81**	**1.7%**
*At4g13620*		-	-		
*At4g06746*	*RAP2.9*	-	-		
*At5g21960*		-	-		
*At1g71520*		-	-		
*At1g63040*		-	-		
*At2g40350*	*DREB2H*	-	-		
*At2g40340*		-	-		
*At5g11190*	*SHN2*	-	-		
*At4g27950*		-	-		
*At1g49120*		-	-		
*At1g03800*	*AtERF10*	-	-		
*At1g12890*		-	-		
*At1g12980*	*ESR1/DRN*	-	-		
*At1g24590*		-	-		
*At1g04370*	*AtERF14*	-	-		
*At5g50080*		-	-		
*At5g64750*	*ABR1*	-	-		

The data for microaspirated syncytia at 5 dpi and 15 dpi were compared with control roots (the elongation zone without root tip was used as control). The third and fourth columns show the normalized expression values on a log_2_ scale. The differences (fold changes) between the pairwise samples displayed (fifth column) are accordingly normalized log_2_ ratios (see the Online Methods section for details). The q-values in column 6 indicate significance after correction for multiple testing controlling the False Discovery Rate. Genes with a significant up- or downregulation (false discovery rate < 5%) are shown in bold. A dash (−) indicates that the gene is not represented on the GeneChip.

### Promoter::GUS and qRT-PCR analysis of *RAP2.6* expression in syncytia

**Figure 1:**
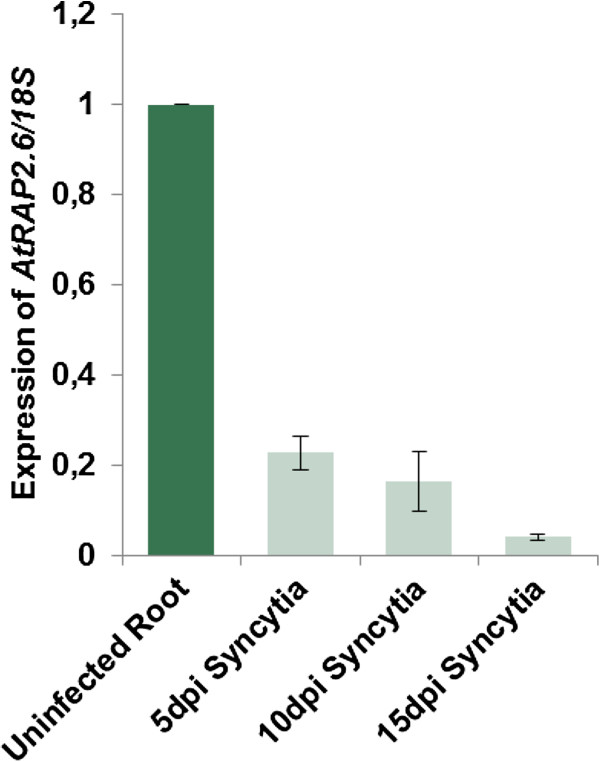
**Expression of *AtRAP2.6 *in response to nematode infection.** Expression of *AtRAP2.6* in wild type plants was determined by qRT-PCR in 5, 10, and 15 dpi syncytia and uninfected root segments (containing elongation zones without root tips from 15-d-old seedling). The data included three independent biological and three technical replicates. Values are means ± SE, n = 3. The bar shows standard error for the mean.

The expression of *RAP2.6* in syncytia was studied by using qRT-PCR for which syncytia were excised at 5, 10 and 15 dpi. For comparison with the GeneChip results, the same control root segments were used as in that study [16]. *RAP2.6* was highly downregulated in syncytia compared with controls at all time points (Figure 1), thus validating the GeneChip data which also showed strong suppression of this gene in syncytia.

**Figure 2:**
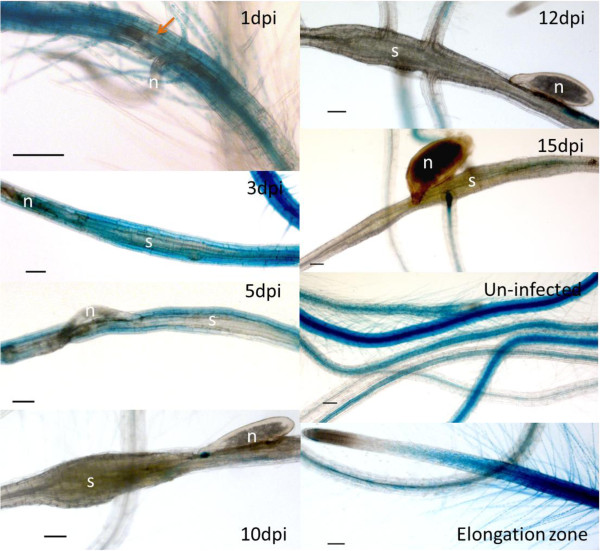
**GUS expression in syncytia.** GUS staining of a prom*RAP2.6*::GUS line was performed for 1, 3, 5, 10, 12, and 15 dpi syncytia. Arrow shows cells differentiating into a syncytium. N = nematode, S = syncytia and bar = 100 μm.

To further study the expression of *RAP2.6* in syncytia, a prom*RAP2.6*::GUS fusion was constructed in pMAA-Red [33] and used to transform Arabidopsis plants. A representative homozygous promoter::GUS line was infected with second stage juveniles under sterile conditions and stained for the detection of GUS activity at different time-points after infection, i.e. 1, 3, 5, 10, 12 and 15 dpi along with uninfected roots (Figure 2). GUS expression was seen in the vicinity of the nematode infection site at 1 dpi but had disappeared in the ISC. At 3 and 5 dpi, expression was switched off in most of the feeding sites but GUS staining was still visible in the cells surrounding the syncytia. At 10, 12, and 15 dpi, GUS expression was neither found in syncytia nor in cells surrounding the syncytia. On the other hand, highly intense expression was found in the uninfected roots of promoter*RAP2.6*::GUS plants in 20-d-old plants (corresponding to 5 dpi), which was then confined to younger root parts and lateral roots in 25- and 30-d-old roots (corresponding to 10 and 15 dpi). The promoter*RAP2.6*::GUS analysis also confirmed the GeneChip data.

**Figure 3:**
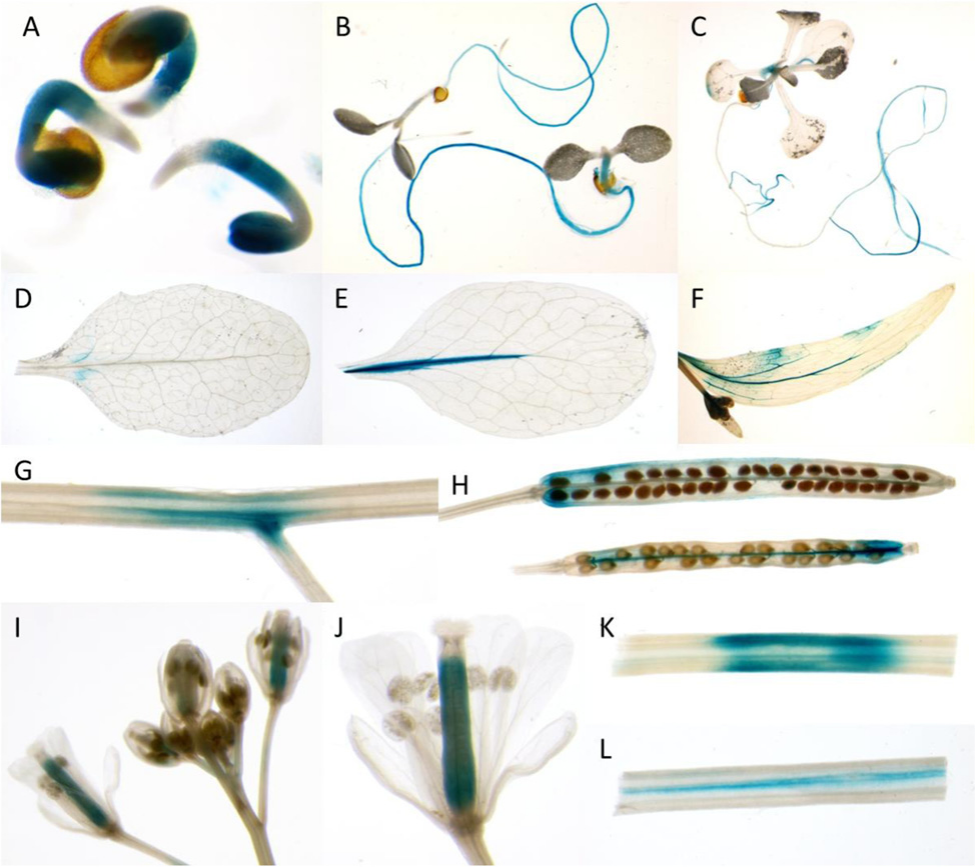
***RAP2.6 *promoter activity determined with GUS fusions. A**, 1-d-old seedlings, **B**, 5-d-old seedlings, **C**, 14-d-old seedlings, **D**, 5- w-old rosette leaf, **E**, rosette leaf after flowering, **F**, cauline leaf, **G**, stem with point of secondary branches, **H**, siliques, **I**, inflorescence, **J**, flower, **K**, stem, **L**, longitudinal section of stem. The arrow in I points to the replum which showed GUS staining in younger siliques.

**Figure 4:**
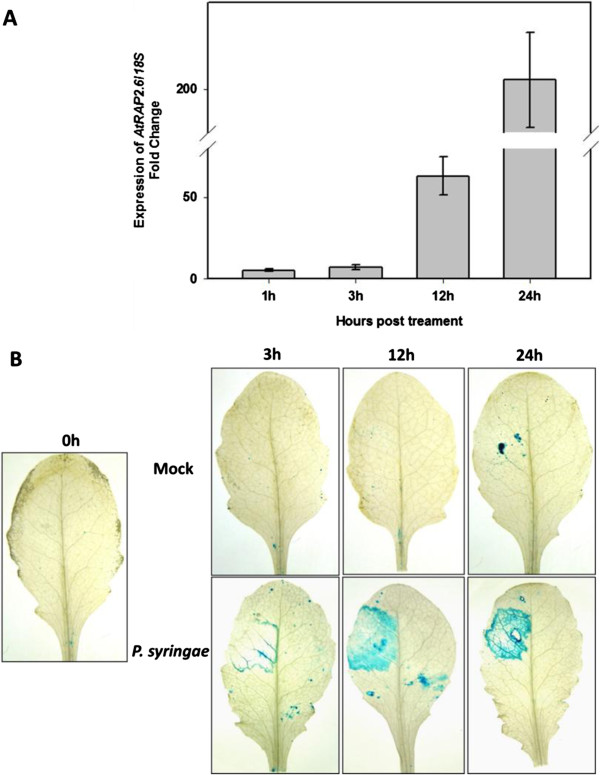
**Expression of *RAP2.6 *in response to *P. syringae *pv tomato DC3000. A**, Expression of *RAP2.6* in wild type plants in response to *P. syringae* pv tomato DC3000 was determined by qRT-PCR. The data included three independent biological and three technical replicates. Values are means ± SE, n = 3. The bar shows standard error for the mean. **B**, GUS staining of rosette leaves of a prom*RAP2.6*::GUS line after mock infiltration (MgCl_2_) and infiltration with *P. syringae* pv tomato DC3000 at different time points.

**Figure 5:**
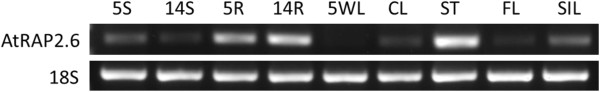
**developmental regulation of *RAP2.6.*** RT-PCR using RNA isolated from seedlings grown on MS-medium (5S, 5-d-old shoots; 14S, 14-d-old shoots; 5R, 5-d-old roots; 14R, 14-d-old roots) or from plants grown on soil (5WL, 5-w-old leaves; CL, cauline leaves, ST, stems; FL, flowers; SIL, siliques). Primers for the 18S gene were used for control reactions.

A prom*RAP2.6*::GUS line has been reported before, however, the authors showed only pictures for seedlings, flowers, and a siliqua [24]. We have therefore included here a developmental analysis of our line (Figure 3). GUS staining in 1-d-old seedlings was observed in cotyledons and roots but not root tips. In 5-d-old seedlings promoter activity was found in roots but not root tips and in the hypocotyl. Cotyledons at this stage did not show GUS staining. A similar result was found for 14-d-old seedlings but older roots were not stained. No staining was found in older rosette leaves, except some small very weak patches in some leaves (compare also Figure 4). After flowering, a staining was found in the main leaf vein. Cauline leaves also showed staining in leaf veins and a weak patchy staining. We also detected GUS staining in stems, especially in the vasculature. In flowers the GUS expression was confined to the carpels and in young siliques staining was mainly found in the replum while older siliques only showed some GUS expression in parts of the valves. GUS expression was confirmed by RT-PCR (Figure 5) which detected the strongest *RAP2.6* expression in roots and stems. GUS expression after infiltration of *P. syringae* pv *tomato* DC3000 (Figure 5) also confirmed the report of [25,27] that *RAP2.6* is induced by *P. syringae*.

### Overexpression lines and mutants of *RAP2.6*

**Figure 6:**
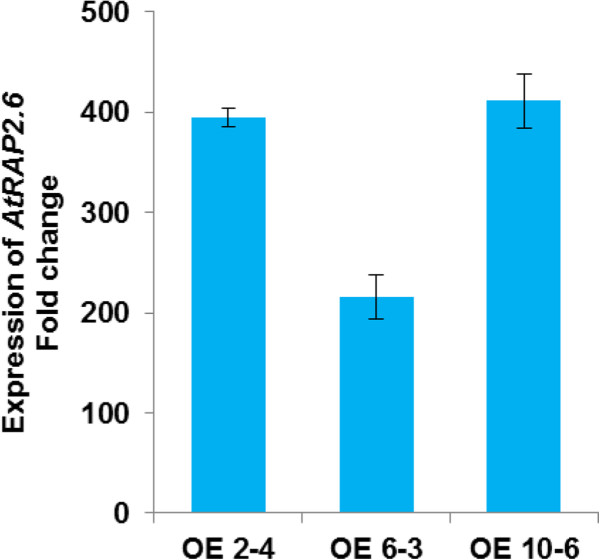
**Overexpression of *RAP2.6 *in pMAA-Red.** Transgenic lines #2, 6, 10 were selected based on RT-PCR of seedlings to measure the transcript level quantitatively by qRT-PCR.

Several reports have shown that *RAP2.6* was involved in resistance responses [25,27,29,30]. This indicated that nematodes might downregulate the expression of *RAP2.6* to avoid resistance responses of the plant. We therefore produced overexpression lines using the vector pMAA-Red. The selection of homozygous lines was made first by visual observation based on the degree of DsRed fluorescence in seeds of different lines as described [33]. Three lines which showed strong fluorescence were made homozygous followed by qRT-PCR and compared to wild type using 18S as an internal control (Figure 6). The 14-d-old seedlings of selected overexpression lines showed a much higher transcript level as compared to wild type (Col). As has been reported before [24], RAP2.6 overexpression resulted in early flowering but the phenotype of seedlings was not different from wild type in our assays (data not shown).

**Figure 7:**
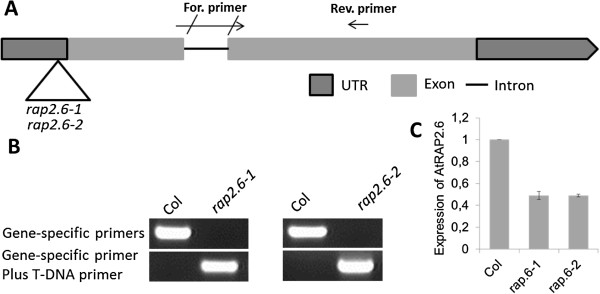
**Knock-out mutants for *RAP2.6*. A**: The T-DNA insertion (inverted triangle) in *rap2.6-1* (GK_053G11 .01) and *rap2.6-2* (GK_053G11 .02) is located in the 5´UTR. **B**: PCR with DNA from homozygous mutants and Columbia (Col). **C**: qRT-PCR for measurement of the expression of mutants as compared with WT in 14-d-old seedlings. For. primer and Rev. primer indicate the positions for the forward and reverse primer used for qRT-PCR.

For *RAP2.6* one knock-out mutant (GK-053G11) with several T3 seed lines was available. The insertion of the T-DNA is in the 5´untranslated leader region (Figure 7A). The T-DNA insertions of two T3 lines were confirmed by PCR (Figure 7B) as described in Material and Methods. The transcript level of homozygous lines was measured by qRT-PCR which showed that *RAP2.6* was approximately 50% downregulated in seedlings (Figure 7C). The downregulation of *RAP2.6* in these mutants resulted in late flowering of the mutant plants but otherwise the phenotype of seedlings was not different from wild type seedlings (data not shown).

### Overexpression of *RAP2.6* has no effect against *Pseudomonas syringae*

**Figure 8:**
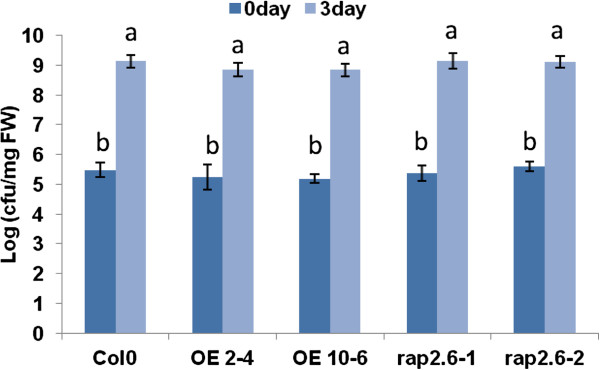
**Infection assay with *P. syringae *pv tomato DC3000.** Overexpression lines, mutant, and wild type control were infected by dipping. Data were analysed for significance difference using ANOVA (P < 0.05) and LSD. Values are means ± SE.

As *RAP2.6* was reported to be highly activated by virulent and avirulant strains of *P. syringae*[25,27] which we confirmed by analysis of a promRAP2.6::GUS line (Figure 5), we also tested the effect of overexpression or mutants of *RAP2.6* on the pathogenic strain *P. syringae* pv *tomato* DC3000. Neither overexpression nor knocking out of *RAP2.6* had an effect on the growth of *P. syringae* pv *tomato* DC3000 as compared to wild type plants (Figure 8). This suggested that *RAP2.6* is not involved in resistance or susceptibility against *P. syringae* pv *tomato* DC3000.

### Overexpression of *RAP2.6* results in resistance against nematodes

**Figure 9:**
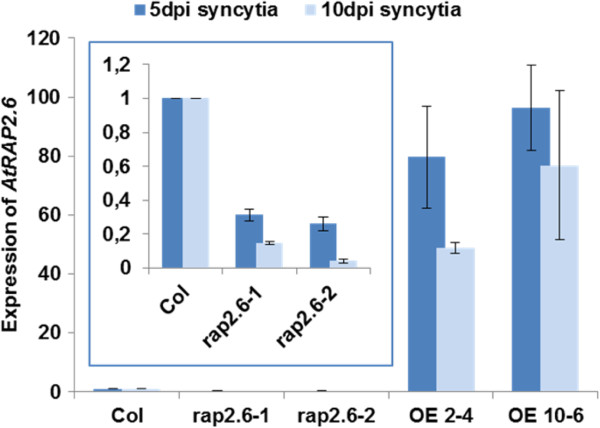
**Expression of *RAP2.6 *in syncytia of overexpression lines and mutants.** Syncytia at 5 and 10 dpi where cut out from the roots and total RNA was isolated. Expression of *RAP2.6* was measured by qRT-PCR. The data included three independent biological and three technical replicates. Values are means ± SE, n = 3.

Since expression in seedlings might be different from expression in syncytia, we determined the expression level of *RAP2.6* in syncytia of mutant lines and overexpression lines (Figure 9). Compared to wild type plants, expression in syncytia at 5 and 10 dpi was much lower in both mutant lines but strongly upregulated in overexpression lines. In general, the expression was lower in 10 dpi syncytia.

**Figure 10:**
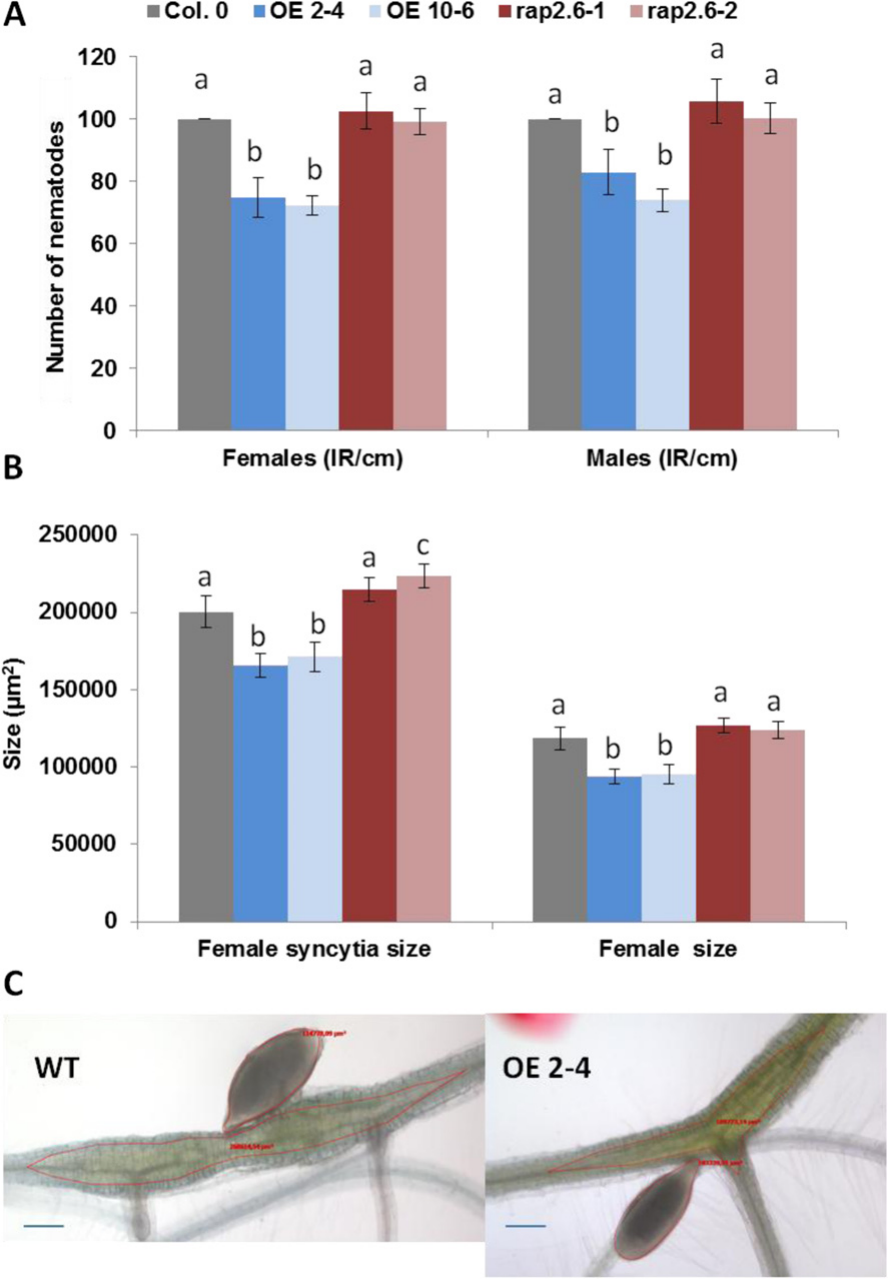
**Nematode resistance test.** The resistance of overexpression lines and knock-out mutants of *RAP2.6* was compared to wild type plants after infection with *H. schachtii*. **A**: Number of male and female nematodes per cm of root length calculated at 15 dpi setting the wild type as 100%. The statistical significance was determined by three independent replicates. Values are means ± SE, n = 15. The bar shows standard error for the mean and different letters indicate significante differences (P < 0.05; ANOVA and LSD). **B**: Size of female syncytia and female nematodes at 14 dpi. Ten syncytia were selected randomly from three independent replicates (total = 30) and the size of syncytia and associated female nematodes was determined. Data were analysed for significance difference using ANOVA (P < 0.05) and LSD. Values are means ± SE. **C**: Representive pictures of the photographed syncytia and nematodes from wild type Col and overexpression line OE 2–4. Scale bar (blue) = 100 μm.

We performed nematode infection assays with two overexpression lines and the two mutant lines and compared the results to wild type plants (Figure 10). Both overexpression lines supported a significantly lower number of female and male nematodes as compared to the wild type. The overexpression lines also resulted in impaired development of syncytia associated with female nematode and female nematodes which were smaller as compared to those from wild type plants. However, the T-DNA insertion mutants did not show significant differences from wild type in terms of number of female and male nematodes or size of female nematodes. Only the size of syncytia associated with female nematodes was affected by this mutation and was significantly larger for line *rap2.6-2*.

### Nematode resistance in overexpression lines might be regulated by SA and JA pathways

**Figure 11:**
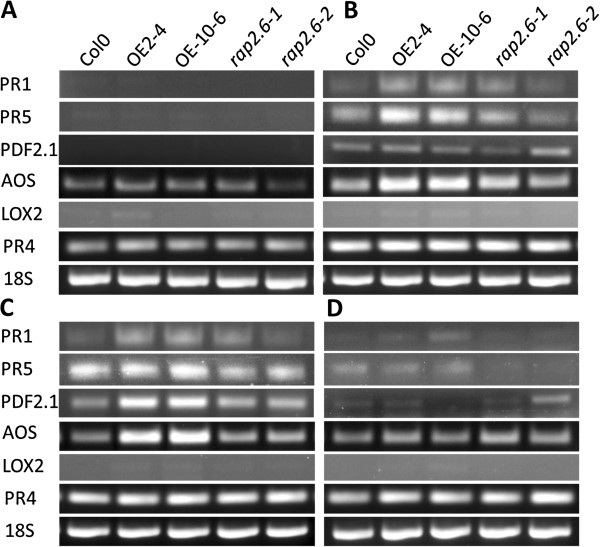
**Expression of different JA and SA inducible genes in overexpression lines and mutant in response to nematode infection. A**: uninfected root, **B**: 1 dpi root segments, **C**: 2 dpi root segments and **D**: 5 dpi syncytia.

The roots of overexpression and mutant lines along with wild type Col were infected with J2 larvae in 2 independent experiments. RNA was isolated from uninfected, 1 dpi and 2 dpi roots along with 5 dpi syncytia. The expression of marker genes which are induced by ethylene (ET) (*PR4*), SA (*PR1* and *PR5*), JA/ET (*Pdf2.1a*), and JA (*AOS* and *LOX2*) was determined by RT-PCR in these samples (Figure 11). Uninfected wild type together with overexpression lines and mutants showed similar expression in uninfected roots with low levels of *AOS* and *PR4* and no expression of *PR1*, *PR5*, *Pdf2.1a*, and *LOX2* (Figure 11A). However, SA inducible genes *PR1* and *PR5* were slightly induced at 1 dpi in overexpression lines as compared with wild type (Col) and mutants which indicated that the initial responses of the plant might be regulated by SA (Figure 11B). Similarly, the JA-inducible gene *AOS* showed upregulation at 1 dpi in overexpression lines which was more prominent than *PR1* and *PR5*. On the other hand, *Pdf2.1a*, *LOX2* and *PR4* showed no upregulation in either overexpression lines or mutants and as compared to wild type (Figure 11B). At 2 dpi, most of the genes showed the same expression as in 1 dpi except *Pdf2.1a* which was induced in overexpression lines (Figure 11C). In 5 dpi syncytia, expression of all tested genes was similar to the wild type in the overexpression lines and the mutant lines (Figure 11D). Compared to uninfected controls, nematode infection led to an early plant response which was indicated by upregulation of *PR1*, *PR5*, *Pdf1.2a*, *AOS*, and *PR4*.

### Callose deposition is enhanced in RAP2.6 overexpression lines

**Figure 12:**
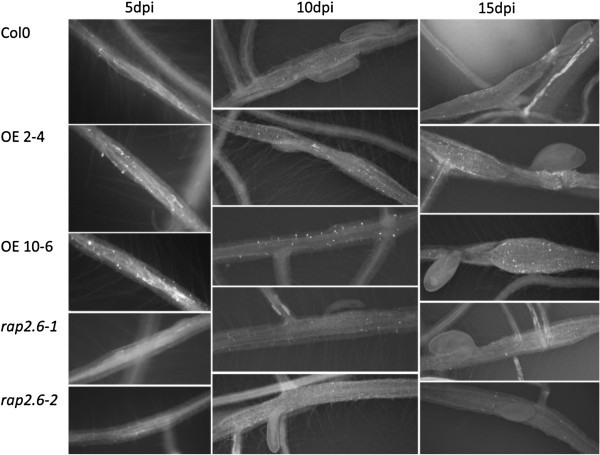
**Callose deposition in syncytia.** Callose staining of syncytia of wild type, overexpression lines, and mutant lines at 5, 10, and 15 dpi. Representative pictures are shown.

**Figure 13:**
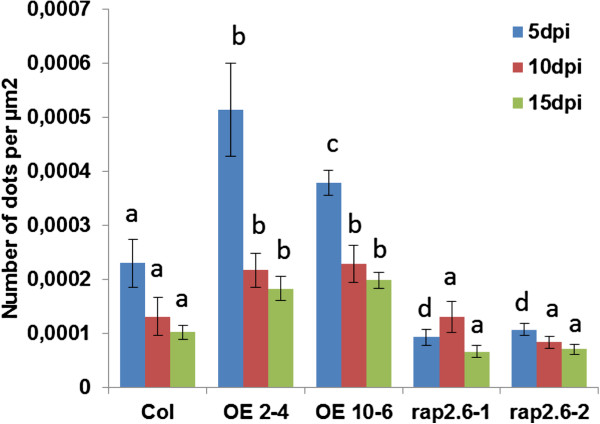
**Quantification of callose deposition.** Syncytia of wild type, overexpression lines, and mutant lines at 5, 10, and 15 dpi were stained for callose. The area of syncytia was measured and the number of dots within the area was counted. The data are the mean from 10 syncytia. Data were analysed for significance difference using ANOVA (P < 0.05) and DMRT. Values are means ± SE.

We performed callose staining of 5, 10 and 15 dpi syncytia which showed that the overexpression lines accumulated more callose as compared to the mutants and wild type plants at all the time points. Representative pictures are shown in Figure 12. Callose deposits were more prominent and higher in number in the feeding sites of overexpression lines. Quantification of the number of dots confirmed the visual observation (Figure 13). The number of callose deposits at the feeding sites was significantly higher in the overexpression lines at all time points as compared to wild type while the mutant lines had a significantly lower number of deposits.

## Discussion

### ERF genes are preferentially downregulated in syncytia

Of the 105 ERF genes included on the Arabidopsis GeneChip, 32 were significantly downregulated in syncytia induced by *H. schachtii* in Arabidopsis roots while only 7 were significantly upregulated. Besides *RAP2.6*, the downregulated genes included *RAP2.4* (also named *WIND1*), which is involved in the wound response [34] and *At3g50260* (*DEAR1*), a positive regulator of cell death and PR-gene expression [32]. Another downregulated gene was *ORA59*[31], which is involved in the JA-regulated resistance response. Furthermore, the transcription factors ERF5, ERF6 and to some extent ERF8 have been reported to be involved in chitin-induced resistance reactions of Arabidopsis [35]. The genes for ERF5 and ERF6 were both significantly downregulated in syncytia. Thus, downregulated ERF genes included those for transcription factors important for the expression of resistance-related genes, supporting the observation that *H. schachtii* downregulates the resistance response in syncytia.

A notable exception was *RAP2.3*, which was expressed in roots and syncytia at a very high level and which was even more upregulated in syncytia as compared to roots. However, it has been shown that RAP2.3 functioned as a suppressor of cell death in yeast [36] and its upregulation in syncytia would therefore be important to support the development of syncytia. RAP2.3 was not the focus of this work but it might be interesting to study its role for syncytium development in detail.

### Expression of *RAP2.6*

The starting point for this work was the observation that the *RAP2.6* gene was strongly downregulated in syncytia as determined by a transcriptome analysis of syncytia [16]. We have confirmed this downregulation by qRT-PCR of syncytia cut out from infected roots and by analysis of a prom*RAP2.6*::GUS line. The expression of *RAP2.6* has been studied before. According to Genevestigator [37] (Additional file 3) this gene is especially expressed in protoplasts and in roots. The strongest expression in roots was in the maturation zone. Expression in inflorescences and especially rosette leaves and seedlings was found by qRT-PCR [24]. These authors also produced promoter::GUS fusions which showed expression in roots of 7-d-old seedlings, petals, carpels, and the valves of immature siliques. Our promoter::GUS line confirmed the expression in seedlings and carpels but we did not find expression in petals and the expression in the valves of siliques was weak. The reason for these differences is not known and might be related either to the promoter fragment or the specific GUS lines that were used. However, all our results, including the GUS analysis after the induction by *P. syringae* pv tomato DC3000 and the analysis of GUS expression after infection with *H. schachtii* are in line with previous observations [16,25,27] and our qRT-PCR results.

### Transcriptional response of *RAP2.6* to different stimuli/stresses

Induction of *RAP2.6* by both JA and SA has been demonstrated [23-25]. Similarly, activation of this gene in response to ABA and various abiotic stresses such as salt, heat, drought, and osmotic stress has been reported [23,24]. In addition to these stresses, it seemed that *RAP2.6* is also inducible by wounding as indicated by our GUS analysis of induction after *P. syringae* pv tomato DC3000 infiltration where some GUS staining was observed at 24 hpi of mock infiltration.

### Role of *RAP2.6* for nematode development

Cyst nematodes manipulate the expression of various plant genes which leads to the development of syncytia as their sole nutrient source [38]. Defense-related genes are preferentially downregulated [16,17] which might be achieved through the activity of effectors produced by the nematode and injected into the syncytium. Several recent reports support this hypothesis. Expression of the putative *H. glycines* effector Hg30C02 in Arabidopsis increased susceptibility to *H. schachtii* possibly by interfering with a plant PR-protein [39,40]. Furthermore, it has recently shown that *Globodera rostochiensis* produces an effector (SPRYSEC-19) which is able to suppress plant defense responses [41]. It is justified to assume that nematodes produce a variety of effectors (suppressors) that are involved in downregulating defense-related genes in syncytia [42]. Among such downregulated genes in syncytia [16] were for instance *WRKY33* ([43], Ali et al., manuscript in preparation) and *RAP2.6*. *RAP2.6* belongs to the large family of ethylene response factors. Many of these are transcription factors which respond to ethylene or JA stimuli. Another example is for instance *ORA59*[31] which is also downregulated in syncytia [16].

Overexpression of *RAP2.6* resulted in higher resistance against *H. schachtii*, supporting the consideration that downregulation of *RAP2.6* in syncytia is important for compatibility. The T-DNA mutant *rap2.6* did not show an effect in our resistance assays except a small effect on syncytium size. In case of *P. syringae*, bacteria are still able to induce JA-dependent pathways, thus suppressing the SA pathway which leads to a compatible interaction. In case of *H. schachtii*, the downregulation of *RAP2.6* by the nematode is obviously sufficient to completely block downstream resistance reactions and therefore the mutant did not further enhance the susceptibility.

The analysis of JA-, SA-, and ET-responsive genes indicated that the nematodes might induce an initial plant response during early infection resulting in the induction of SA-, JA-, and ET-dependent plant resistance responses. This response was elevated in the overexpression lines with the strongest enhancement found for JA-inducible genes. These results indicated that the enhanced resistance found against *H. schachtii* might be the result of JA-dependent reaction mechanisms. Induction of PR genes in Arabidopsis roots after *H. schachtii* infection has been reported before [40]. It has been suggested that cyst nematodes suppress SA-dependent resistance at their feeding sites [44]. The JA pathway was found to be important for resistance of rice against root knot nematodes [45] but nothing is known about JA-dependent signaling in Arabidopsis roots infected with *H. schachtii* except that defense-related genes including those dependent on JA-signaling are downregulated in syncytia [16]. At the moment we do not know for sure if the enhanced resistance of RAP2.6 overexpression lines is a consequence of the expression of JA-dependent defense genes or due to enhanced callose deposition (or both). However, considering that the induction of JA-dependent defense genes was only found at very early time points makes it very likely that the enhanced callose deposition was the main reason for the resistance of the overexpression lines.

Callose deposition is known as a plant resistance response to invading pathogens [46]. This reaction is also increasingly used to quantify the reaction to bacterial PAMPs (Pathogen Associate Molecular Patterns) such as flagellin (see for instance [47]. Callose deposition is also one of the earliest plant responses to invading nematodes [48,49]. The degradation of callose deposited outside of the cell membranes in the plant roots is important for nematode development [50]. However, nothing is known about the role of callose in plant resistance against nematodes although it could be imagined that callose might be used to plug the plasmodesmata between syncytia and phloem cells [51]. It is for instance known that resistant rice plants plug the sieve plates with callose in response to feeding of the brown planthopper [52].

## Conclusion

Our results showed that overexpression of RAP2.6 led to enhanced callose deposits in syncytia. Callose deposition at syncytium plasmodesmata would disturb nutrient import into syncytia and would inhibit the development of the nematodes since these are dependent on nutrients supplied through syncytia. It would therefore be interesting to further explore the role of callose in resistance against cyst nematodes in more detail.

## Methods

### Plant cultivation

Arabidopsis (ecotype Columbia) plants were grown in soil in growth chambers at 25°C in long day conditions (16 h light / 8 h dark). For growth in sterile conditions, seeds were surface sterilized for 7 min in 10% (w/v) sodium hypochlorite and subsequently washed three times with sterile water. Seeds were placed in Petri dishes (9 cm) on a modified Knop medium with 2% sucrose [53] or on MS medium containing 3% sucrose [54].

### Production of promoter::GUS and overexpression lines

The promoter region 1333 bp upstream the start codon of the *RAP2.6* gene (*At1g43160*) was amplified by PCR (Phusion High-Fidelity DNA Polymerase from Thermo Scientific) using 50 ng Arabidopsis Columbia genomic DNA as template. The primer pair used for amplification of the promoter region were promRAP2.6forEcoRI and promRAP2.6revNcoI (Additional file 4). Primers included restriction sites for EcoRI and NcoI for subsequent cloning into the binary vector pMAA-Red [33]. This plasmid harbors the DsRed gene for plant selection. It also contains the double enhanced 35S promoter of the cauliflower mosaic virus (CaMV) and TMV omega element as translational enhancer fused to the GUS reporter. During the cloning procedure the 35S promoter was exchanged by the promoter fragment of *RAP2.6*. For construction of overexpression lines a cDNA clone for *RAP2.6* (RIKEN, Japan, http://www.riken.go.jp) was used as template. The cDNA was amplified by PCR using Phusion polymerase with RAP2.6forBspHI and RAP2.6revBamHI primers (Additional file 4). The primers included the BspHI and BamHI restriction sites for subsequent cloning into the binary vector pMAA-Red, this time replacing the GUS gene.

The promoter::GUS and overexpression constructs were introduced into *Agrobacterium tumefaciens* GV3101 for transformation of Arabidopsis plants by the floral dip method [55]. The fluorescent transformed seeds were selected under an inverse microscope equipped with a DsRed fluorescence filter (Axiovert 200M; Zeiss AG, Germany) and put on soil to grow the next generation. Homozygous lines were selected based on visual observation as described [33].

### Mutant screening

Two independent lines from a single knockout mutant of RAP2.6 were obtained from the Arabidopsis stock center (GK_053G11.01 with stock number N301757 for *rap2.6-1* and GK_053G11.02 with stock number N301758 for *rap2.6-2*) (Figure 6). These are individual T3 seed lines for the parental line GK-053G11. The DNA of different segregating plants of each line was isolated [56] and PCR analysis (Gk-Lb primer and primer pairs used for screening of single mutants are shown in Additional file 4) was used to identify homozygous knockouts.

### Nematode infection assays

*H. schachtii* cysts were harvested from *in vitro* stock cultures propagated on mustard (*Sinapsis alba* cv Albatros) roots growing on 0.2 concentrated Knop medium supplemented with 2% sucrose [53]. The cysts were soaked in 3 mM ZnCl_2_ as stimulus for hatching of J2 larvae under sterile conditions. The J2 larvae were then washed three times in sterile water and resuspended in 0.5% (w/v) Gelrite (Duchefa, Haarlem, The Netherlands) before inoculation. Twelve-d-old Arabidopsis roots were inoculated under sterile conditions with about 50–60 juveniles per plant. At 14 dpi, pictures of female syncytia and female nematodes (longitudinal optical sections) were taken using an inverse microscope (Axiovert 200M; Zeiss AG, Germany). The syncytia and females were outlined using the Axiovision Kontour tool (Zeiss AG, Germany) and the area was determined by the software. Afterwards, the number of males and females per cm of root length was counted at 15 dpi. Root length was scored according to [57] by comparing the roots growing on agar plates with pictures for the different classes of root growth. The data regarding number of nematodes and sizes of nematodes and syncytia were analysed using single factor ANOVA (P < 0.05). As the F-statistic was greater than F-critical, a Least Significance Test (LSD) was applied.

### GUS analysis

Histochemical detection of GUS activity was performed by staining using X-gluc (Biomol, Hamburg, Germany) in 0.1 M sodium phosphate buffer pH 7.0, 0.1% Triton-X 100, 0.5 mM K_3_[Fe(CN)_6_], 0.5 mM K_4_[Fe(CN)_6_] and 10 mM Na_2_EDTA. For GUS staining of syncytia, the infected roots (infection was done as described above) of promRAP2.6::GUS plants were incubated with X-gluc overnight at 37°C. The staining was examined at 1, 3, 5, 7, 10, and 15 dpi. Stained syncytia and uninfected roots were photographed under an inverse microscope (Axiovert 200M; Zeiss, Hallerbergmoos, Germany) having an integrated camera (AxioCam MRc5; Zeiss).

### RNA isolation

Plant samples were immediately frozen in liquid nitrogen. Total RNA was isolated using a NucleoSpin® RNA Plant kit (genXpress) according to the manufacturer’s instructions, including DNase digestion. However, this DNase treatment did not completely digest the DNA present in the sample. For some experiments the remaining DNA was therefore digested using Ambion® DNA-free™ DNase Treatment and Removal Reagents (Invitrogen). RNA was quantified using NanoDrop (NanoDrop™ 2000c from PEQLAB). Isolated RNA was stored immediately at −80°C.

### Reverse Transcriptase (RT-PCR) and quantitative Real Time PCR (qRT-PCR)

RT-PCR was done using the RT-PCR Master Mix (USB) following the manufacturer’s instructions. For cDNA synthesis Superscript III reverse transcriptase (Invitrogen) and random primers (oligo(dN)6) according to the manufactures instructions were used. The qRT-PCR was performed on an ABI PRISM 7300 Sequence Detector (Applied BioSystems). Each qRT-PCR sample contained 12.5 μl Platinum SYBR Green qPCR SuperMix with UDG and ROX (Invitrogen), 2 mM MgCl_2_, 0.5 μl forward and reverse primer (10 μM), 2 μl cDNA and water to make a 25 μl total reaction volume. The primer pairs used for *RAP2.6* were RAP2.6qRTfor and RAP2.6qRTrev which are given in Additional file 4. Control reactions with no cDNA template ruled out false positives. Dissociation runs were performed to make sure that there was no formation of primer dimers. The 18S gene was used as an internal reference. Results were calculated using the Sequence Detection Software SDS v2.0 (Applied BioSystems). Relative expression was calculated by the (1 + E)^-ΔΔCt^ method [58].

### Callose staining of syncytia

Nematode infection of wild type, overexpression lines, and knockout mutants was carried out as described above. At 5, 10, and 15 dpi syncytia were stained for callose deposition as described by Millet et al. [47] with some modifications. The syncytia were fixed in a 3:1 ethanol:acetic acid solution for 4 h. The fixative was changed after two hours for thorough fixing and clearing of the tissues for good callose detection. Syncytia were rehydrated in 30% ethanol for 3 h and water overnight. After three water washes, seedlings were treated with 10% NaOH and placed at 37°C for 1 h to make the tissues transparent. After four water washes, the syncytia were incubated at room temperature in 150 mM K_2_HPO_4_, pH 9.5, and 0.01% aniline blue (Sigma-Aldrich) for 2 hours. The callose was observed immediately using an inverse microscope (Axiovert 200M; Zeiss, Hallerbergmoos, Germany) with integrated camera (AxioCam MRc5; Zeiss) under UV (excitation, 390 nm; emission, 460 nm). The callose deposition was quantified per unit area basis. For this the area of the syncytia was measured and dots within the area were counted.

### *Pseudomonas syringae* infection assay

The infection assay was carried out according to Tornero and Dangl [59] with some modifications using the pathogenic strain *Pseudomonas syringae* pv tomato DC3000. Approximately 24 h prior to inoculation, and in order to obtain a lawn of bacteria, a bacterial inoculum was distributed onto fresh King's B-medium plates and incubated for 24 h at 28°C. Then, 15 ml of 10 mM MgCl_2_ was added to the plates to scrape of the bacterial lawn and resuspended in a falcon tube. A bacterial pellet was obtained after centrifugation at 4000 rpm for 10 min and resuspended again in 10 mM MgCl_2_. The bacterial suspension was diluted to an OD_600_ of 0.05 with 10 mM MgCl_2_ and silwet was added to a final concentration of 200 μl/L. Pots with Arabidopsis plants (15-d-old seedlings) were then turned upside down, dipped in the bacterial suspension and swirled for 10 seconds. After infection, the plants were covered with a transparent lid and moved back to the growth chamber.

One hour after the inoculation, and for each investigated line, around 50–100 mg of infected seedlings (only aerial parts) were transferred into a pre-weighed 1.5 ml tube containing 200 μl of 10 mM MgCl_2_ and 200 μl/L silwet. The tubes were shaken (250 rpm) in a 2 litre Erlenmeyer for one hour at 28°C. After that, 20 μl from each tube were added to a 96-well plate containing 180 μl of 10 mM MgCl_2_ (without silwet). By using a multichannel pipette, serial 10-fold dilutions from the bacterial suspension were prepared, spotted onto fresh King'sB medium plates and incubated for 24 hours at 28°C. The numbers of colonies were counted to determine the colony forming unit (CFU) per unit fresh weight. The CFU data (log_10_) for 0 dpi and 3 dpi were calculated using the CFU equation given by Tornero and Dangl [59].

For GUS staining, the promRAP2.6::GUS line was grown on soil in short day conditions and after 5 weeks rosette leaves were mock infiltrated with MgCl_2_ or infiltrated with *P. syringae.* The staining was done at 0, 3, 12 and 24 h with X-Gluc for 8 hours at 37°C.

### Statistical analysis of microarray data

Affymetrix CEL files from Szakasits et al. [16] were analyzed using packages of the Bioconductor suite (http://www.bioconductor.org). For details see Szakasits et al. [16]*.* For the statistical tests, individual gene variances have been moderated using an Empirical Bayes approach as described in Siddique et al. [17] and in the online methods (Additional file 5). Tests were restricted to the subset of 105 genes of the 122 ERF group genes that could be probed on the GeneChip, with the group as defined before [20] and containing the originally described RAP2 proteins as different subgroups. This considerably increases the statistical power of the testing procedure as it reduces the necessary correction for otherwise massive multiple testing.

## Competing interests

The authors declare that they have no competing interests.

## Authors’ contributions

MAA carried out most of the experiments and helped writing the manuscript. AA did the *Pseudomonas* experiments. DK did the bioinformatics analysis. HB conceived of the study, and participated in its design and coordination, was involved in analyzing the experiments and writing the manuscript. All authors read and approved the final manuscript.

## Supplementary Material

Additional file 1 MA plot (syncytium vs. root) for ERF genes. Click here for file

Additional file 2 MA plot (15 dpi syncytium vs. 5 dpi syncytium) for ERF genes. Click here for file

Additional file 3 Gene expression of RAP2.6 according to Genevestigator. Click here for file

Additional file 4 Primers used in this work. Click here for file

Additional file 5 Online methods bioinformatic analysis. Click here for file
