# Supplementary material for: Overexpression of the transcription factor RAP2.6 leads to enhanced callose deposition in syncytia and enhanced resistance against the beet cyst nematode Heterodera schachtii in Arabidopsis roots
Source: BMC Plant Biol. 2013 Mar 19;13:47. doi: 10.1186/1471-2229-13-47 (PMC3623832; doi:10.1186/1471-2229-13-47)
Supplement: Additional file 2 — MA plot (15 dpi syncytium vs. 5 dpi syncytium) for ERF genes. [file 1471-2229-13-47-S2.docx]

**Additional file 2 – MA plot (15 dpi syncytium vs. 5 dpi syncytium) for ERF genes.**
