# Supplementary figures and images for: Overexpression of the transcription factor RAP2.6 leads to enhanced callose deposition in syncytia and enhanced resistance against the beet cyst nematode Heterodera schachtii in Arabidopsis roots
Source: BMC Plant Biol. 2013 Mar 19;13:47. doi: 10.1186/1471-2229-13-47 (PMC3623832; doi:10.1186/1471-2229-13-47)

**Additional file 3 – Gene expression of *RAP2.6* according to Genevestigator.**


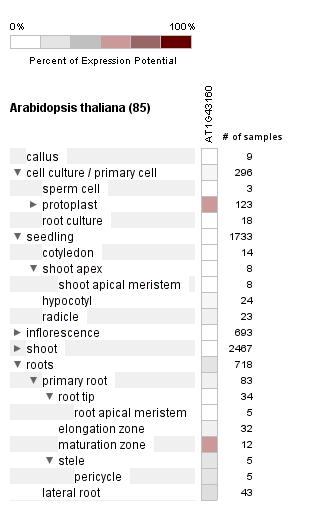

Supplement: Additional file 3 — Gene expression of RAP2.6 according to Genevestigator. [file 1471-2229-13-47-S3.docx]
