# Supplementary material for: Overexpression of the transcription factor RAP2.6 leads to enhanced callose deposition in syncytia and enhanced resistance against the beet cyst nematode Heterodera schachtii in Arabidopsis roots
Source: BMC Plant Biol. 2013 Mar 19;13:47. doi: 10.1186/1471-2229-13-47 (PMC3623832; doi:10.1186/1471-2229-13-47)
Supplement: Additional file 4 — Primers used in this work. [file 1471-2229-13-47-S4.docx]

**Additional file 4 – Primers used in this work**

| **Name** | **Sequence** | **Amplification** |
| --- | --- | --- |
| RAP2.6forBspHI | TTTCAATCATGATGTCTATGCT | RAP2.6 coding sequence |
| RAP2.6revBamHI | ACCAATTGATTGTATCCTAGGTGCG |  |
| prom RAP2.6forEcoRI | ACGACATATCCAATAACACAATTTG | RAP2.6 promoter sequence |
| prom RAP2.6revNcoI | ACACCATGGTTGAAATTGC |  |
| RAP2.6qRTFor | CCGGTTCAGCTGTGACTAAAG | *RAP2.6* qPCR |
| RAP2.6qRTRev | CTGAGTTCCAACATTTTCGGG |  |
| GK-LB | ATATTGACCATCATACTCATTGC | Mutant screening |
| GK_053G11 .01 LP | TGATGTCGGCAGTTACAAGTG | *rap2.6-1* mutant screening |
| GK_053G11 .01 RP | TTCCTCTAAAGCGAAGTGCTG |  |
| GK_053G11 .02 LP | TATGGGCATCGAAAAACAGAC | *rap2.6-2* mutant screening |
| GK_053G11 .02 RP | AGATGCGTAGACATGTAGCATG |  |
| PR1For | GTGACTTGTCTGGCGTCTCC | *PR1* |
| PR1Rev | ACGTGTGTATGCATGATCACATC |  |
| PR4For | CTGGACCGCCTTCTGCGGG | *PR4* |
| PR4Rev | AGCCTCCGTTGCTGCATTGGT |  |
| PR5For | TCACATTCTCTTCCTCGTGTTC | *PR5* |
| PR5Rev | GATGGTCTTATCCCCAGCTTG |  |
| PDF1.2aFor | GCTAAGTTTGCTTCCATCATCACC | *Pdf1.2a* |
| PDF1.2aRev | GTGTGCTGGGAAGACATAGTTGC |  |
| AOS For | GCTTCTATTTCAACCCCTTTTCC | *AOS* |
| AOS Rev | ACGGTCTTTGATTGGTCCTAC |  |
| LOX2For | GAAACAAGTCTTCACGCCAG | *LOX2* |
| LOX2Rev | CTTATCTTCCTCAGCCAACCC |  |
| 18SFor | GGTGGTAACGGGTGACGGAGAAT | *18S* |
| 18SRev | CGCCGACCGAAGGGACAAGCCGA |  |
